# Treatment of adult metachromatic leukodystrophy model mice using intrathecal administration of type 9 AAV vector encoding arylsulfatase A

**DOI:** 10.1038/s41598-021-99979-2

**Published:** 2021-10-15

**Authors:** Noriko Miyake, Koichi Miyake, Atsushi Sakai, Motoko Yamamoto, Hidenori Suzuki, Takashi Shimada

**Affiliations:** 1 Department of Biochemistry and Molecular Biology, Nippon Medical School, 1-1-5 Sendagi, Bunkyo-ku, Tokyo, 113-8602 Japan; 2 Department of Gene Therapy, Nippon Medical School, Tokyo, 113-8602 Japan; 3 Department of Pharmacology, Nippon Medical School, Tokyo, 113-8602 Japan

**Keywords:** Preclinical research, Molecular medicine, Paediatric research

## Abstract

Metachromatic leukodystrophy (MLD) is a lysosomal storage disease caused by an arylsulfatase A (ARSA) deficiency and characterized by severe neurological symptoms resulting from demyelination within the central and peripheral nervous systems. We investigated the feasibility and efficacy of intrathecal administration of a type 9 adeno-associated viral vector encoding ARSA (AAV9/ARSA) for the treatment of 6-week-old MLD model mice, which are presymptomatic, and 1-year-old mice, which exhibit neurological abnormalities. Immunohistochemical analysis following AAV9/ARSA administration showed ARSA expression within the brain, with highest activities in the cerebellum and olfactory bulbs. In mice treated at 1 year, alcian blue staining and quantitative analysis revealed significant decreases in stored sulfatide. Behaviorally, mice treated at 1 year showed no improvement in their ability to traverse narrow balance beams as compared to untreated mice. By contrast, MLD mice treated at 6 weeks showed significant decreases in stored sulfatide throughout the entire brain and improved ability to traverse narrow balance beams. These findings suggest intrathecal administration of an AAV9/ARSA vector is a promising approach to treating genetic diseases of the central nervous system, including MLD, though it may be essential to begin therapy before the onset of neurological symptoms.

## Introduction

Metachromatic leukodystrophy (MLD) is an autosomal recessive inherited lysosomal storage disorder caused by a deficiency in the lysosomal enzyme arylsulfatase A (ARSA), which catalyzes the degradation of galactosyl-3-sulfate ceramide (sulfatide), a major myelin sphingolipid^1^. This disease is characterized pathologically by myelin degeneration in both the central and peripheral nervous systems (CNS and PNS)^2^. Clinically, the disease manifests as progressive motor and mental deterioration that is ultimately lethal^3^. MLD is classified into three or four different forms based on the patient’s age at onset^4,5^. Late infantile is the most frequently observed and severe type^6^. These patients usually develop neurological symptoms that include gait disturbance and loss of speech at around 2 years of age and die within a few years after appearance of the first symptoms^1^. Although enzyme replacement therapy using human ARSA has been tried, this approach does not effectively relieve the neurological symptoms (NCT00681811; http://www.clinicaltrials.gov). A novel therapy that can stop or reverse the progression of this neurological disorder is therefore needed.

Gene therapy is one of the potentially effective strategies under consideration for use in the treatment of CNS disorders, and several gene therapy protocols for treating MLD have been proposed^7^. However, the blood brain barrier (BBB), which limits the delivery of systemically administered therapeutic molecules to the brain, is a major obstacle to successful treatment of any neurological disorder affecting the CNS^8,9^. Consequently, how to deliver a therapeutic gene across the BBB and then how to distribute it to the entire brain are crucial challenges that must be overcome. One simple strategy is direct injection of viral vectors into the brain^10,11^; another is hematopoietic stem cell gene therapy^12,13^. A clinical trial of lentiviral vector-mediated hematopoietic stem cell gene therapy has been conducted in presymptomatic MLD patients and provides strong evidence of clinical benefit (NCT015601821).

We recently succeeded in treating neonatal ARSA knockout MLD model mice through systemic gene delivery of a single-strand type 9 adeno-associated viral vector encoding human ARSA (AAV9/ARSA)^14^. Unfortunately, however, intravenous systemic gene delivery is generally much less effective in adult mice than neonatal mice, though Audouard et al. recently reported full reversal of symptoms in the same models following intravenous AAV delivery^15^. We also previously showed in ARSA knockout mice that intrathecal injection of a type 1 AAV vector encoding ARSA into a single site within the cisternal space results in widespread gene delivery to the brain and dorsal root ganglia (DRG)^16^. Other groups have also demonstrated the utility of intrathecal delivery of type 9 AAV for transduction within the CNS^17–19^, and this strategy has been used to treat the lysosomal storage diseases MPS I and Pompe disease^20,21^.

In the present study, we used a type 9 AAV vector previously shown to efficiently transduce the CNS^22–24^ and tested the feasibility and potential efficacy of intrathecal administration of AAV9/ARSA for the treatment of MLD in adult mice. In addition, to determine whether overt neurological symptoms could be reversed by the gene therapy, we also assessed the effect of AAV9/ARSA in 1-year-old MLD mice, which already exhibited the neurological symptoms of the disease. Because AAV gene therapy for MLD has yet to be tested intrathecally, and human trials have been limited to ex vivo lentiviral-mediated hematopoietic stem cell gene therapy^12,13^ and intracerebral injection of AAV in MLD patients^25^, it is important to examine the effect of intrathecal administration of AAV9/ARSA for the treatment of MLD.

## Results

### Intrathecally injected AAV9/GFP is efficiently transduced into the cerebellum, brainstem, spinal cord and DRG

**Figure 1:**
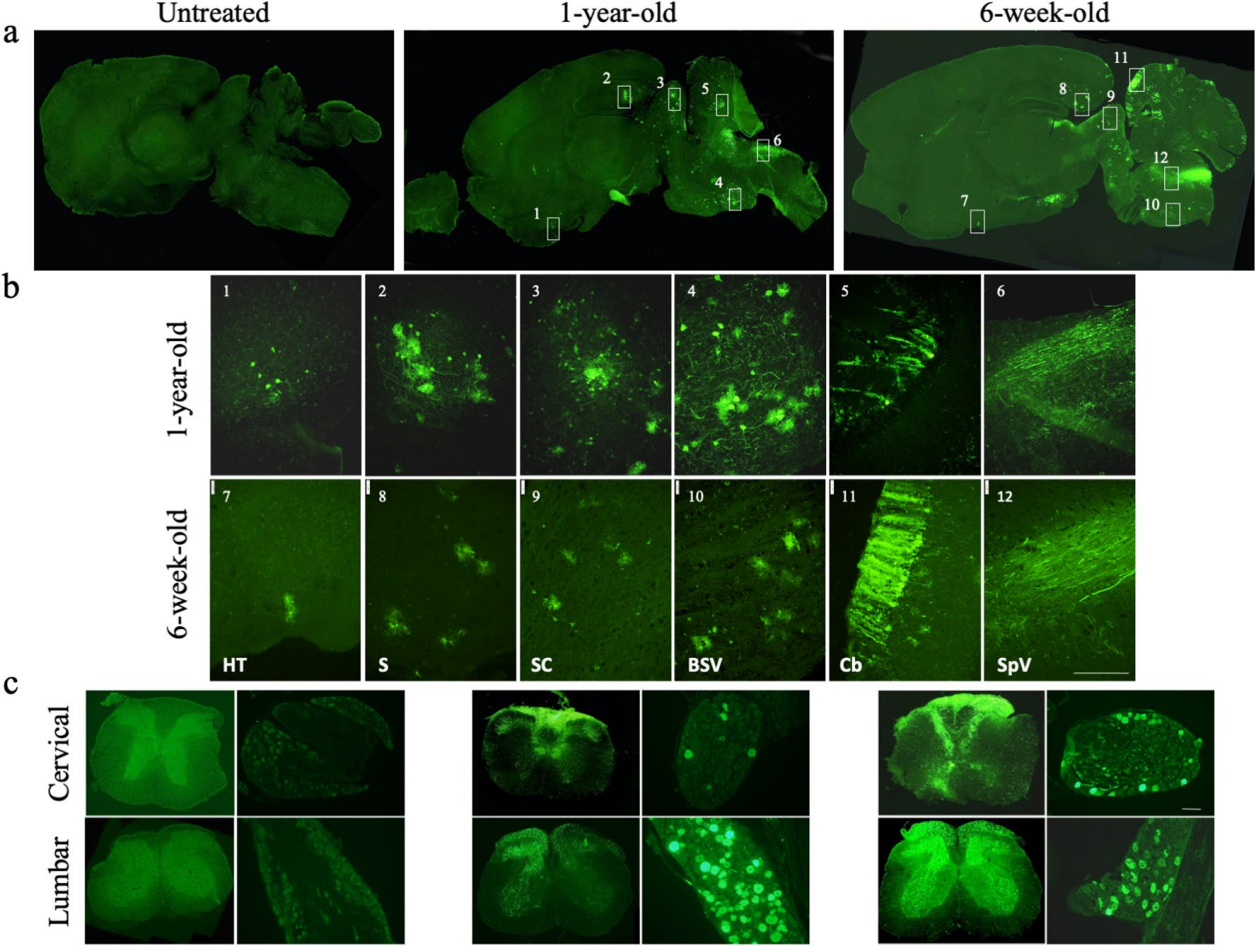
Expression of GFP after intrathecal injection. AAV9/GFP was intrathecally injected via suboccipital puncture into 1-year-old and 6-week-old MLD mice. Representative images of immunostaining for GFP in sagittal sections of brain (**a**), spinal cord and dorsal root ganglia (DRG) (**c**). Boxes indicate the approximate regions from which the higher magnification images shown in (**b**) were acquired. (**b**) 1and 7, hypothalamus (HT); 2 and 8, subiculum (S); 3 and 9, inferior colliculus (IC); 4 and 10, brain stem ventral (BSV); 5 and 11, cerebellum (Cb); 6 and 12, spinal trigeminal tract (SpV). Sections were taken 3 or 13.5 months after injection of AAV9/GFP. Bar: 200 μM.

We first analyzed the transduction efficiency and distribution of an AAV9 vector encoding green fluorescent protein (AAV9/GFP: 4.0 × 10^11^ vg) in 1-year-old (n = 5) and 6-week-old MLD (n = 4) mice following intrathecal injection via a suboccipital puncture. We decided the dosage of AAV vector based on previous papers^24,26^. Because ARSA enzyme may be expressed in non-transduced cells by cross collection^27^, we used GFP to confirm only purely transduced cells. The distribution of AAV9/GFP in the brain, spinal cord and DRG was then determined 3 or 13.5 months after the injection through immunohistochemical detection of GFP. Efficient GFP expression was detected in the midbrain, brainstem, cerebellum (Fig. 1a,b) and both the cervical and lumbar spinal cord (Fig. 1c). In addition, a large number of nerve fibers in the dorsal spinal cord and numerous neuronal cell bodies in the DRG were also transduced (Fig. 1c). These results may indicate that DRG neurons are efficiently transduced by intrathecal injection of AAV9 vector and that GFP is transported via ascending fibers in the dorsal spinal cord.

### Efficient ARSA expression in the cerebellum and spinal cord

**Figure 2:**
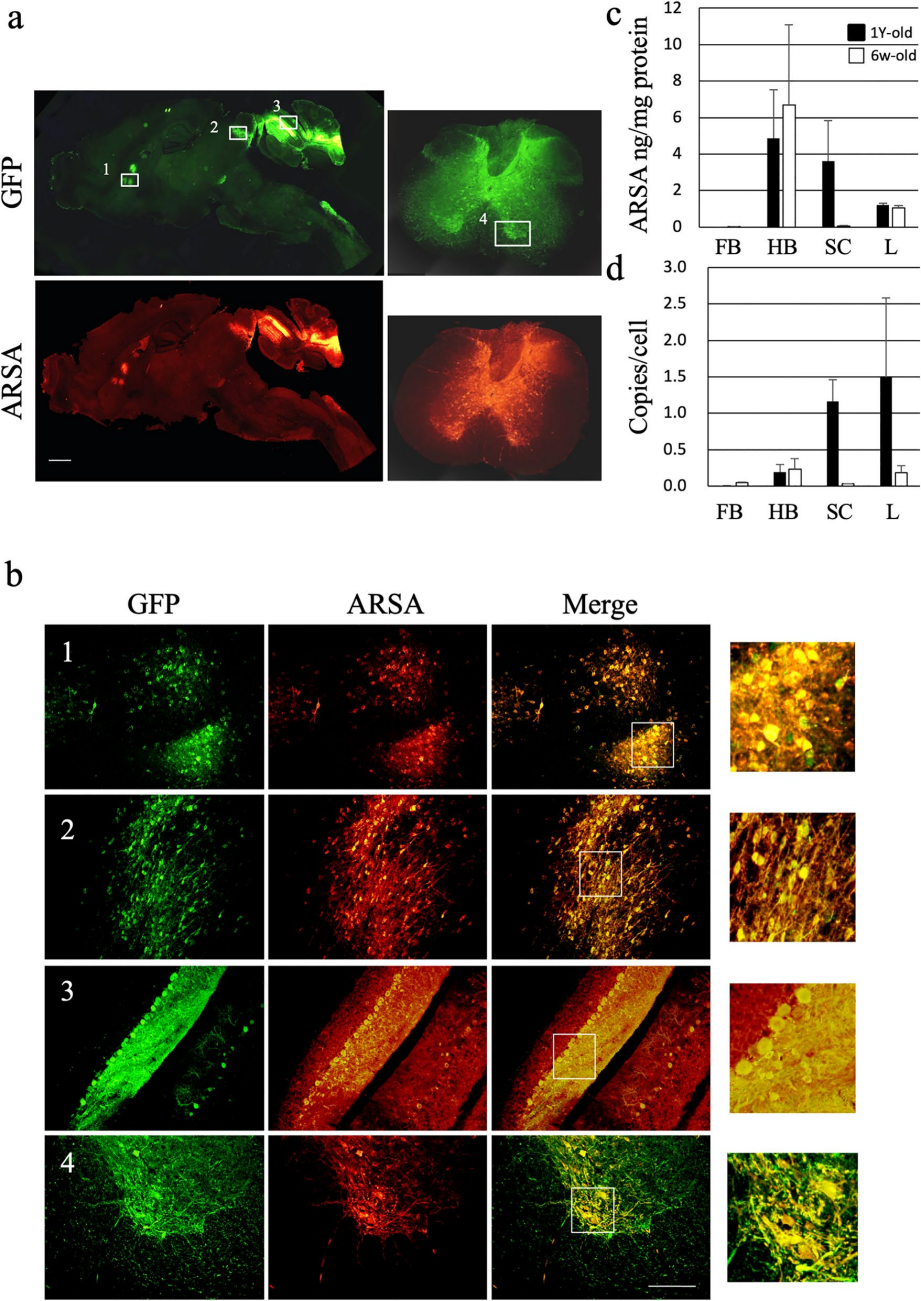
Expression of GFP and ARSA in the brains of treated MLD mice. AAV9/ARSA (encoding ARSA and GFP) were intrathecally injected via suboccipital puncture into 1-year-old MLD mice. (**a**) representative images of immunostaining for GFP and ARSA in sagittal brain and spinal cord sections taken 3 months after injection of AAV9/ARSA. Boxes indicate the approximate regions from which the higher magnification images shown in **b** were acquired. (**b**) 1, caudate putamen (striatum); 2, inferior colliculus; 3, cerebellum; 4, ventral horn of the cervical spinal cord. Bar: 200 μM. Panels on the right highlight the observed co-localization. (**c**) ARSA levels in the indicated brain regions measured with ELISAs in MLD mice treated at 6 week (n = 4) and 1 year (n = 5) of age. (**d**) droplet digital PCR to detect the copy numbers of AAV vector in MLD mice treated at 6 week (n = 4) and 1 year (n = 5) and of age. FB, forebrain; HB, hindbrain; SC, spinal cord; L, liver; ARSA, human arylsulfatase A.

We next treated MLD mice using an AAV9 vector encoding ARSA together with GFP (AAV9/ARSA). These vectors were intrathecally injected into 6-week-old MLD mice (n = 4), which were presymptomatic, and 1-year-old MLD model mice (n = 5), which already showed neurological symptoms. 13.5 months or 3 months after injection, when both groups of mice were around 15 months old, we used immunostaining to assess expression of ARSA and GFP. Figure 2a,b shows the results of intrathecal injection of AAV9/ARSA into 1-year-old MLD mice. Efficient ARSA expression was detected throughout the GFP-positive areas, with the cerebellum and spinal cord showing the highest expression (Fig. 2b). For MLD mice treated at 1 year and 6 week, we also used ELISAs to detect levels of ARSA (Fig. 2c) and the vector genome in the forebrain, hindbrain, spinal cord and liver (Fig. 2d). ARSA protein and the vector genome were detected in hindbrain (treated at 1 year: 4.88 ± 2.66 ng/mg and 0.20 ± 0.10 copy/cell; treated at 6 weeks: 6.69 ± 4.40 ng/mg and 0.24 ± 0.14 copy/cell) and spinal cord of MLD mice treated at 1 year (3.60 ± 2.24 ng/mg and 1.16 ± 0.30 copy /cell) but not in the forebrain and spinal cord of MLD mice treated at 6 weeks. ARSA protein and the vector genome were also detected in the liver (treated at 1 year: 1.21 ± 0.11 ng/mg and 1.49 ± 1.09 copy/cell; treated at 6 weeks: 1.05 ± 0.13 ng/mg and 0.18 ± 0.10 copy/cell) but not in muscle and heart. We think that the intrathecal injected AAV vectors leaked into the bloodstream and were transduced into the liver, where they are more likely to be transduced.

**Figure 3:**
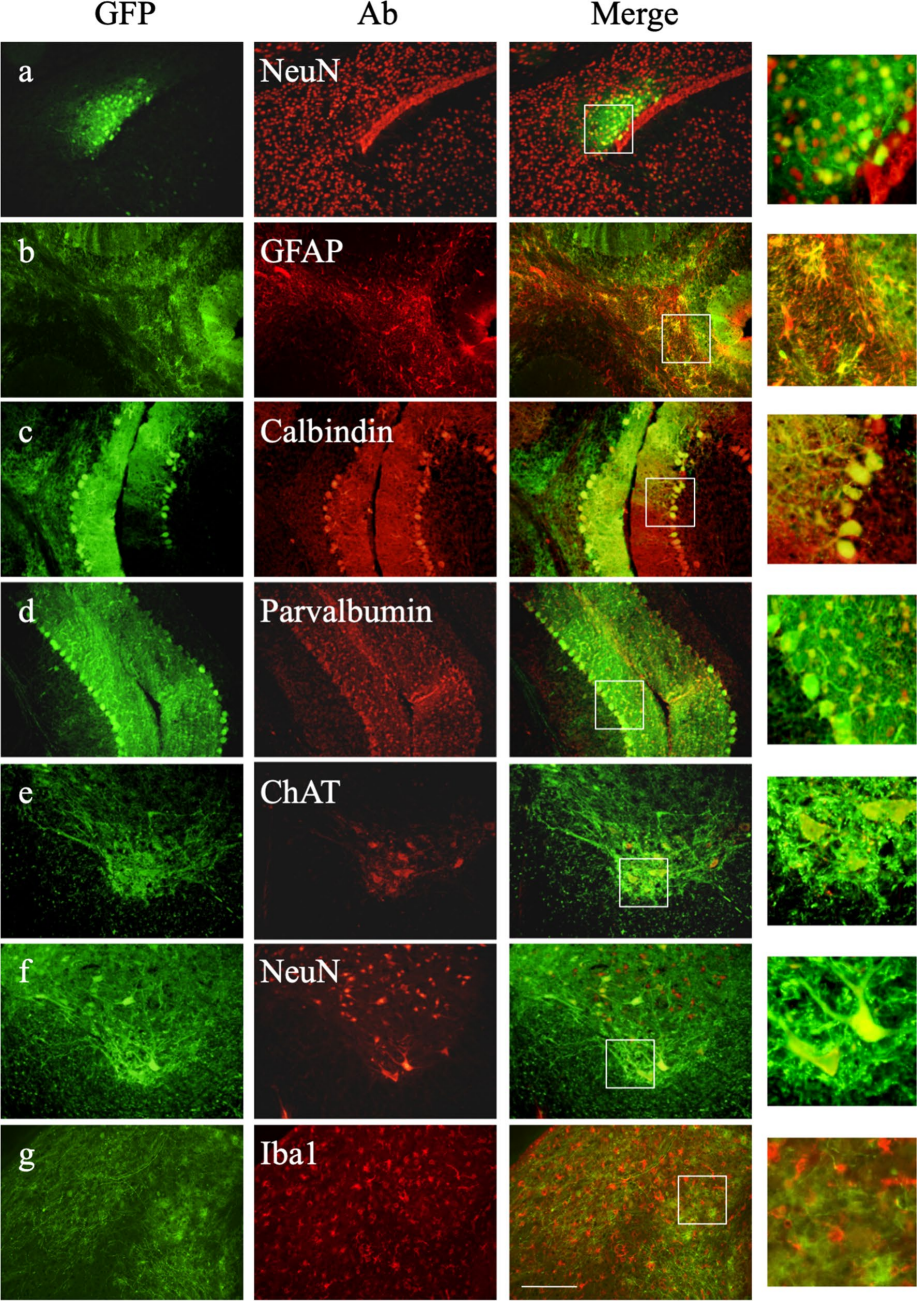
Immunohistochemical analysis of transduced cells in the brain and spinal cord of treated MLD mice at 1 year of age. Immunostaining for GFP and the neuronal marker NeuN (**a**), the astrocyte marker GFAP (**b**), the Purkinje marker calbindin (**c**), the basket marker parvalbumin in the cerebellum (**d**), the microglia marker Iba1 (**g**) in the brain, and ChAT-positive motor neurons (**e**) and NeuN-positive neurons (**f**) in the cervical spinal cord, especially the ventral horn. Bar: 200 μM. Panels on the right highlight the observed co-localization.

Further immunostaining for the appropriate markers showed that GFP-positive cells included NeuN-positive neurons (Fig. 3a), GFAP-positive glial cells (Fig. 3b), calbindin-positive Purkinje cells (Fig. 3c), parvalbumin-positive basket-cells (Fig. 3d) in the brain or choline acetyl transferase (ChAT)-positive motor neurons (Fig. 3e) and NeuN-positive neurons (Fig. 3f) in the cervical spinal cord, especially the ventral horn. On the other hand, GFP-positive cells did not include Iba1-positive microglia (Fig. 3g) in the brain. Nearly the same transduction pattern (transduction into neurons and astrocytes but not microglia) was observed after intrathecal injection into 6-week-old MLD mice (Supplementary Fig. S1). When we checked which type of cells were transduced by intrathecal injection, most of them were neurons and astrocytes as previously reported^23^. The percentages within the brains of treated MLD mice, (calculations were done on random three fields of each mouse (1 year treated: n = 3, 6 week treated: n = 3)) 72.3 ± 5.7% of GFP-positive cells were neurons and 27.3 ± 5.8% of GFP-positive cells were astrocytes in mice treated at 1 year, while 87.3 ± 5.3% of GFP-positive cells were neurons and 12 ± 5% of GFP-positive cells were astrocytes in mice treated at 6 weeks. We detected no GFP-positive oligodendrocytes. Thus, we did not find significant difference between 1-year-old and 6-week-old treated MLD mice.

### Reductions in sulfatide storage and Sulf/GalC ratios in the CNS

**Figure 4:**
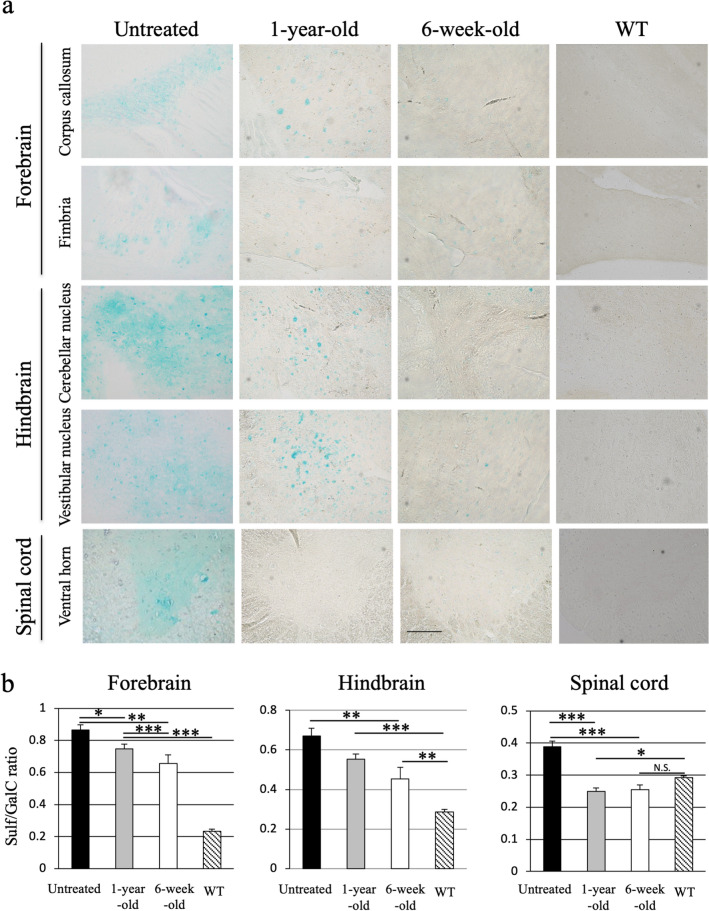
Correction of sulfatide storage in the brain and spinal cord. (**a**) Alcian blue staining of sulfatide in the brain and the spinal cord of 15-month-old MLD mice left untreated or treated with AAV9/ARSA at 1 year or 6 weeks of age. Bar: 200 μM. (**b**) Sulfatide content detected using thin layer chromatography. Ratios of sulfatide (Sulf) to galactosylceramide (GalC) content (Sulf/GalC ratio) in the brain and cervical spinal cord of MLD mice left untreated (closed bars, n = 5), MLD mice treated at 1 year (gray bars, n = 5) or 6 weeks of age (open bars, n = 4), and wild-type (WT) mice (diagonally hatched bars, n = 8). * P < 0.05, **p < 0.01, ***p < 0.0001.

To analyze the potential therapeutic effect of intrathecal injection of AAV9/ARSA into MLD mice, we used alcian blue staining to detect the presence of sulfatides. Sulfatide accumulation within cells was readily detected in 15-month-old untreated MLD mice. Sulfatide was especially detectable in the white matter, forebrain (including the corpus callosum and hippocampal fimbria), hindbrain (cerebellar nucleus and vestibular nucleus) and ventral horn of the spinal cord (Fig. 4a). Notably, MLD mice treated at 1 year or 6 weeks showed markedly less sulfatide accumulation within the brain and spinal cord than did age-matched untreated MLD mice (Fig. 4a). We quantified of sulfatide reduction on alcian blue staining using Image J software (Supplementary Fig. S2).

We also used thin-layer chromatography to quantitatively analyze the abundance of sulfatide in lipid extracts. Sulfatide storage in the brain was evaluated based on the ratio of sulfatide (Sulf) to galactosylceramide (GalC), which is a major component of the myelin sheath in oligodendrocytes. We found that as compared to untreated MLD mice, Sulf/GalC ratios were significantly reduced in the forebrain of MLD mice treated at 1 year or 6 weeks (treated at 1 year: 0.75 ± 0.03, treated at 6 weeks: 0.66 ± 0.05 vs. untreated: 0.87 ± 0.03), in the hindbrain of mice treated at 6 weeks (treated at 1 year: 0.55 ± 0.03, treated at 6 weeks: 0.45 ± 0.06 vs. untreated: 0.67 ± 0.04) and in the cervical spinal cord of mice treated at 1 year or 6 weeks (treated at 1 year: 0.25 ± 0.01 treated at 6 weeks: 0.25 ± 0.02 vs. untreated: 0.39 ± 0.02) (Fig. 4b, Supplementary Table S1). In addition, alcianophilic material was almost entirely absent from the spinal cord, which is nearly identical to what is observed in wild type mice.

**Figure 5:**
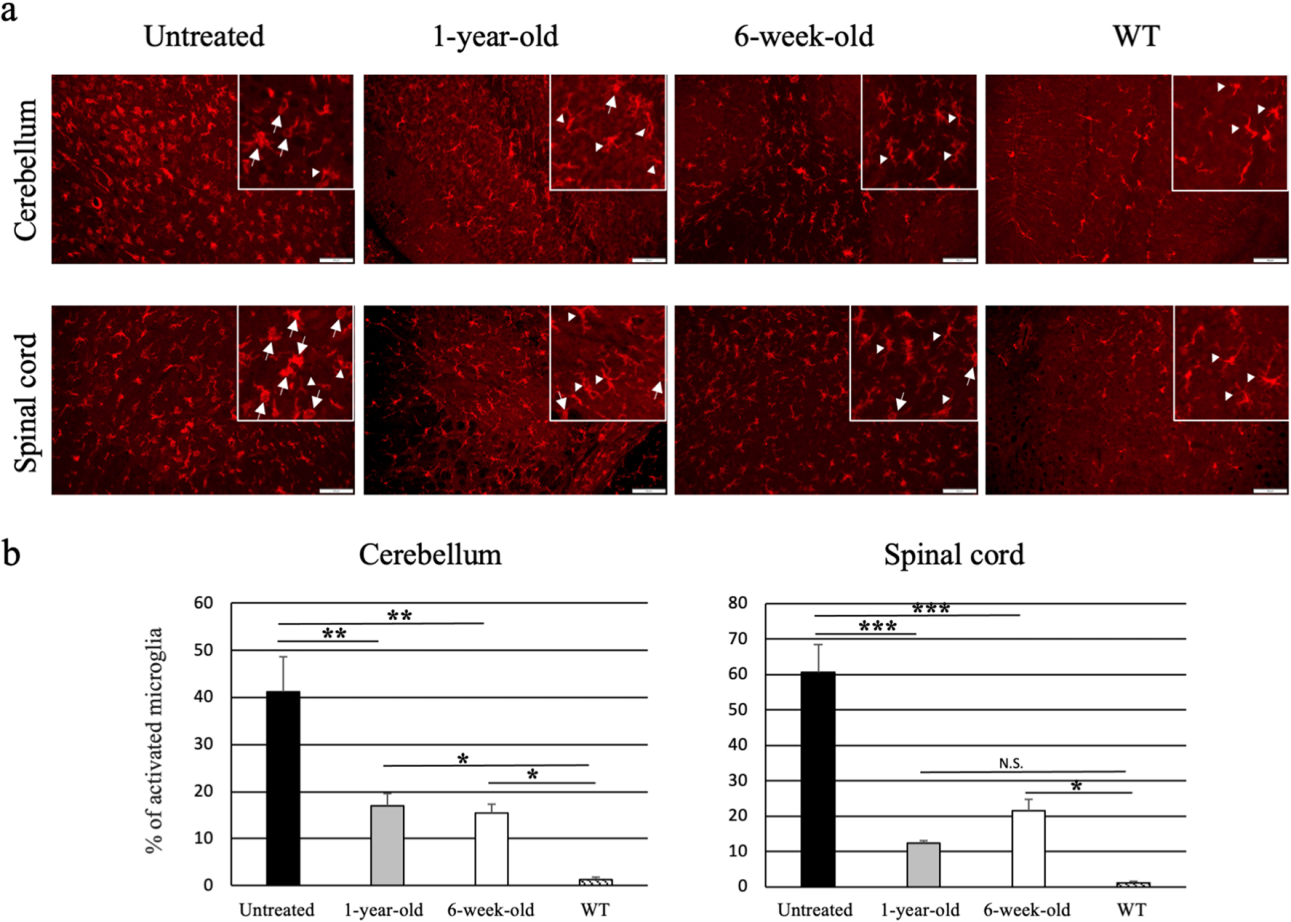
Reduction of activated microglia in AAV9/ARSA-treated MLD mice. (**a**) Immunostaining for the microglia marker Iba1 in the cerebellum and spinal cord. (**b**) Fifteen-month-old untreated mice showed amoeboid microglia cells (arrows), which correspond to activated microglia. MLD mice treated with AAV9/ARSA at 1 year or 6 weeks of age and wild-type (WT) mice showed ramified microglia (arrow heads), which correspond to non-activated microglia. % of activated microglia was calculated as (number of activated microglia/ numbers of Iba1 positive cells). All calculations were done on random three fields of each mouse (untreated: n = 3, 1 year treated: n = 3, 6 week treated: n = 3, and WT: n = 3) Bar: 100 μM. *p < 0.05, **p < 0.01, ***p < 0.0001.

Finally, to assess the effect of AAV9/ARSA treatment on microglial activation, we evaluated staining with anti-Iba1 antibody (Fig. 5a). There was reduction of activated microglia (cells with short projections and large cell bodies; amoeboid microglia) in the both the cerebellum and spinal cord of AAV9/ARSA-treated MLD mice compare to untreated mice (cerebellum: treated at 6 weeks, 15.5 ± 1.85%; treated at 1 year, 17.0 ± 2.6%; untreated, 41.1 ± 7.5%; spinal cord: treated at 6 weeks, 21.57 ± 3.3%; treated at 1 year, 12.3 ± 0.7%; untreated, 60.7 ± 7.9%) (Fig. 5b). Although it is important to analyze molecular markers to verify the expression levels of typical markers of neuroinflammation, including markers discriminating the different states of activation of microglia, microgliosis was improved in treated MLD mice.

### MLD mice exhibit significant improvement in a behavioral test when treated at 6 weeks of age but not at 1 year

**Figure 6:**
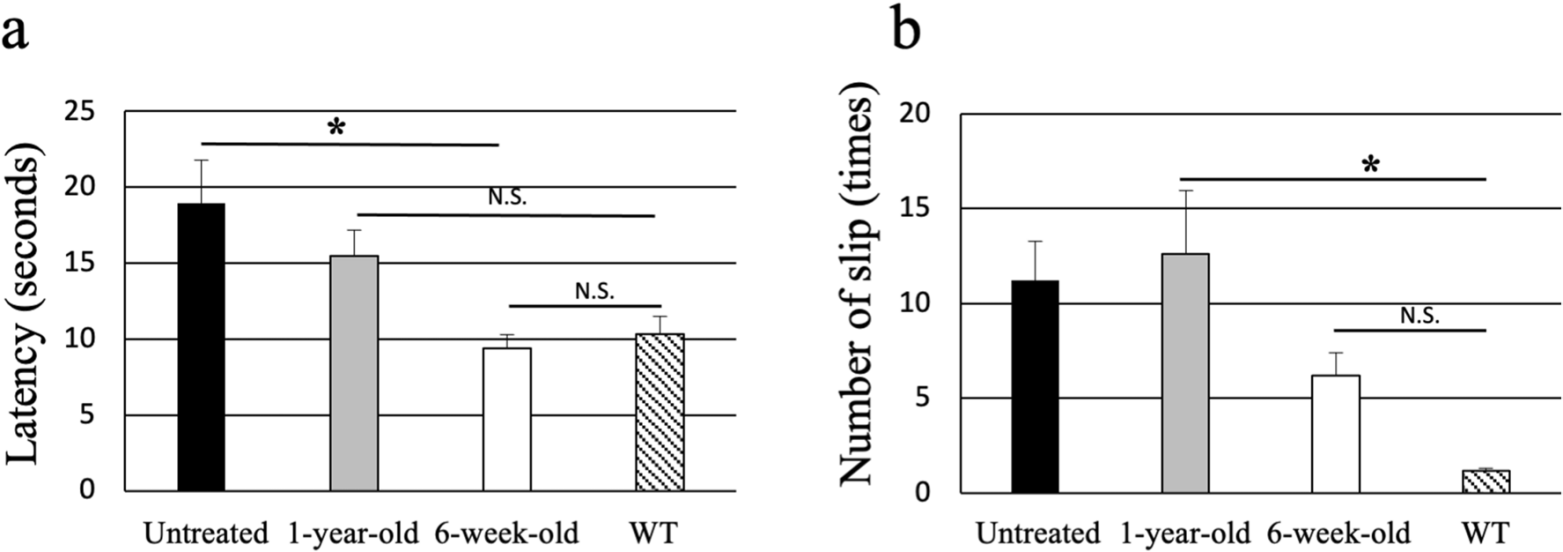
Therapeutic effects of intrathecal injection. Motor function was assessed using a balance beam test with 15-month-old MLD mice left untreated (closed bars, n = 6), treated with AAV9/ARSA at 1 year (gray bars, n = 5), 6 weeks of age (open bars, n = 4) or wild type (WT) (diagonally hatched bars, n = 8). (**a**) Latency: the time required to traverse each beam. (**b**) Slips: the number of times the hind feet of mice slipped off a beam. All animals were given three trials on the beams. *p < 0.03.

To evaluate the effect of intrathecal AAV9/ARSA injection on the neurological symptoms in MLD mice, we compared the abilities of treated and untreated mice to perform a behavioral (balance beam) test as previously described^28^. It was reported that balanced beam test is particularly useful for detecting subtle deficits in motor skills and balance that may not be detected by other motor tests, such as the Rotarod^29^. We found that MLD mice treated at 6 weeks performed better than the untreated mice (latency: 9.4 ± 0.9 s vs. 18.9 ± 2.8 s, p < 0.03; number of slips: 6.2 ± 1.3 vs. 11.2 ± 2.0). Although, the latency of MLD mice treated at 6 weeks was nearly same as that of wild-type mice, the treated MLD mice still exhibited a significantly greater number of slips than wild-type mice. Moreover, MLD mice treated at 1 year showed no significant improvement as compared to the untreated MLD mice (latency: 15.5 ± 1.7 s, number of slips: 12.6 ± 3.4) (Fig. 6).

## Discussion

We previously reported that after intravenous systemic injection of AAV, the vectors were able to pass through the BBB of neonatal mice and efficiently transduce the CNS^30^. Unfortunately, however, systemic administration of AAV vectors is less effective in adult mice than neonatal mice. To address that problem, we tested the utility of intrathecal injection of AAV in adult mice. Following intrathecal injection of an AAV9/GFP vector, GFP expression was seen in the hindbrain and cerebellum. In addition, a large number of nerve fibers in the dorsal spinal cord and numerous neuronal cell bodies in the DRG were also efficiently transduced (Fig. 1). Although, we did no analyze the PNS, Bailey et al. reported that intrathecal delivery of AAV9 resulted in efficient GFP expression in longitudinal sections of sciatic nerve^23^. Thus, intrathecal delivery of AAV9 may be useful for treating the PNS. Although more invasive than an intravenous injection, intrathecal injection may enable use of lower vector doses than are used for intravenous administration. Moreover, with intrathecal injection there is less chance of either germline transduction or generation of neutralizing antibodies than with intravenous administration^17^. Thus, intrathecal injection of AAV vectors could prove highly useful for efficient and long-term expression of desired genes in the CNS and spinal cord and could be an effective means of treating genetic neurological diseases.

In the present study, we clearly demonstrated that intrathecal infusion of AAV9/ARSA into adult MLD mice resulted in efficient gene delivery to the cerebellum, spinal cord and DRG. Overall, however, GFP and ARSA expression were detected in only a limited area of the brain following intrathecal injection of AAV9 vector. Because MLD is a whole brain disease, this limited ARSA expression may not be sufficient to eliminate the MLD disease phenotype. In addition, because MLD is a demyelination disease, we assessed ARSA protein expression in myelin-forming cells. Glial cells positive for both GFAP and ARSA were detected (Fig. 3b), but not microglia positive for both Iba1 and ARSA (Fig. 3g). However, we did not detect any GFP-positive cells among oligodendrocytes. Nevertheless, inhibition of sulfatide accumulation and significantly improved performance of a behavioral (balance beam) test were observed after treatment of 6-week-old mice. One potential explanation for this beneficial effect is the occurrence of “cross-correction,” whereby transduced cells corrected by the gene therapy secrete the transduced lysosomal enzyme, which is then taken up by neighboring cells via the M6P (mannose 6-phosphate) receptor^31^. These successful corrections of biochemical and neurological abnormalities suggest that AAV9-mediated intrathecal gene therapy is a promising approach to treating lysosomal storage diseases with neurological involvement.

Recently, Audouard et al. reported treatment of MLD mice through intravenous injection of AAVPHP.eB carrying human ARSA^15^. The AAVPHP.eB vector passed through the BBB and elicited a full reversal of the symptoms in the same model used in the present study. However, although the AAVPHP.eB vector passed through the BBB in C57BL/6 mice, it did not cross the BBB in BALB/cJ mice^32,33^ or in non-human primates^34^. We therefore need to be cautious about application of this vector for clinical usage. We also did not detect any transduction of oligodendrocytes, major targets of MLD, after intrathecal injection of AAV9/ASA, which would be an important disadvantage of this method. On the other hand, Lattanzi et al*.* demonstrated transduction of oligodendrocytes with a lentiviral vector following intracerebral (external capsule) injection^35^. Similarly, Piguet et al*.* observed transduction of oligodendrocytes with an AAVrh10 vector after intracerebral injection^11^. Use of one of those strategies may be an effective option for transduction of oligodendrocytes. Recently, Rosenberg et al. reported that delivery of ASA through the cerebral spinal fluid is instrumental for ensuring widespread diffusion of the enzyme into the CNS^36^. Although we did not analyze ASA levels in the cerebral spinal fluid, this appears to be an important aspect of intrathecal injection. Moreover, the utility of intrathecal injection of an AAV vector has been demonstrated for the treatment of another lysosomal storage disease, Krabbe disease^37^. That suggests intrathecal injection may be a useful strategy for some lysosomal storage diseases leading to CNS or PNS disorders.

In this study, the area transduced through intrathecal injection was limited to the hindbrain and cerebellum. This is in contrast to the findings of two studies that reported broad gene transfer throughout the brain, including the forebrain, following intrathecal injection^23,26^. We speculate that the reasons for this discrepancy may involve the structure of the AAV vector (single stranded vs. self-complementary, WPRE (−) vs. WPRE ( +)), the injection site (cistern vs. lumbar region), and/or the promoter used (CAG vs CBA promoter). In addition, when Meyer et al. described transduction of the entire CNS in adult nonhuman primates through intrathecal delivery of scAAV9-CBA-GFP, they reported that transduction was more efficient when primates were kept in the Trendelenburg position following administration of the AAV vector^38^. Along the same lines, Bay et al*.* reported holding mice in a tilted 30° head-down position for 6 min as part of the intrathecal injection protocol. Thus, in addition to the route of vector delivery, the position of the animal and the impact of gravity may also be an important factor significantly affecting the distribution of vector^24^. Moreover, because we performed the injection manually, it is possible that variation in the needle depth during the injection resulted in some vector being injected intraparenchymally. This would explain the strong bias for gene expression in brain regions immediately beneath the cisterna magna. It might be helpful to examine the motor cortex in these mice, as specific transduction of cortical motor neurons can occur as a result of retrograde transport following intraparenchymal injection of AAV9 in the hindbrain (beneath the cisterna magna).

To test whether overt neurological symptoms can be reversed by gene therapy, we intrathecally administered AAV9/ARSA to 1-year-old MLD mice, which already exhibit neurological symptoms of the disease. Efficient ARSA expression and a decrease in sulfatides were detected in the hindbrain and spinal cord of mice treated at 1 year. Behaviorally, however, the treated mice showed no significant improvement over untreated control MLD mice, even though the transduction efficiency and ARSA expression did not differ between mice treated at 1 year and 6 weeks. We speculate that significant improvement was observed in mice treated at 6 weeks but not 1 year because ARSA expression after intrathecal injection of AAV/ARSA inhibited further accumulation of sulfatide but did not reduce the amount of sulfatide that accumulated prior to treatment. Alternatively, or in addition, it may be that irreversible histological changes induced by MLD had already occurred by 1 year of age. In addition, although the sulfatide content was lower in treated than untreated MLD model mice, the treatment-induced reduction of sulfatide was limited. The therapeutic effect of a higher dose of vector should therefore evaluated. That said, our findings suggest that for successful treatment of MLD, it is essential to start gene therapy before the onset of neurological symptoms.

Currently ongoing gene therapy for MLD patients entails bone marrow transplantation of ARSA-expressing stem cells transduced with a lentiviral vector^13^. Sevin et al. reported a small trial evaluating direct injection of AAVrh.10-ARSA vector in patients with MLD (NCT01801709). However, this study was terminated due to lack of effectiveness^25^. By contrast, intrathecally injected AAV vector is clearly able to transduce the CNS and mediate ARSA expression in the brain. This strategy may thus be an effective means of treating neurological disorders. At present, however, only limited CNS transduction has been achieved with this approach. It was recently reported that a combination gene therapy entailing direct injection into the brain together with intrathecal injection was more effective than either single treatment alone. The efficacy of such combination therapy has been demonstrated in the Sanfilippo syndrome type B model mouse^39^ and in cases of canine mucopolysaccharidosis VII^40^. In MLD, sulfatide accumulation is detected not only in the CNS, but also in the heart, liver, kidney and other organs. Systemic intravenous injection of AAV9/ARSA would likely decrease sulfatide accumulation in those organs. We previously reported that intraventricular injection of a type 1 self-complementary (sc) AAV vector encoding ARSA suppressed sulfatide accumulation in MLD mice. Noting that Snyder et al. reported the superior transduction properties of AAV6 and 9 for intrathecal injection as compared to AAV1^19^, we used an AAV9 vector in the present study. However, we used a single stranded AAV9 vector, which may have reduced the efficacy after cisterna magna delivery. Combination gene therapy entailing both intraventricular and intrathecal injection of scAAV/ARSA may be a more effective strategy for treating 1-year-old MLD mice and could be potentially useful for treating MLD patients.

We used ELISAs to assess delivery of ARSA and the vector genome to the brain, spinal cord, and liver of intrathecally injected MLD mice. ARSA protein and the vector genome were detected not only in the brain and spinal cord, but also in the liver, which indicates that intrathecally injected AAV/ARSA enters the bloodstream and travels throughout the circulation, reaching the liver. It will therefore be important to analyze the vector distribution in various tissues together with verification of the integration profile of AAV in the CNS and peripheral tissues; and to address concern about potential germline transmission, evaluation of gene transduction of the testis or ovary will be particularly important. Moreover, when assessing the safety of the proposed approach, evaluation of the levels of antibodies against the AAV capsid and the GFP and ASA transgenes in the treated mice were also important. Hordeaux et al. recently observed DRG pathology in 83% of non-human primates administered AAV through the CSF^41^. This DRG toxicity was caused by high transduction rates, which resulted in cellular stress due to an overabundance of the transgene product. That adverse effect could be overcome using microRNA 183^42^. Thus, addressing the DRG toxicity caused by intrathecal injection must be part of future clinical development.

In summary, we have succeeded in treating 6-week-old MLD model mice through intrathecal injection of AAV9/ARSA. However, significant improvement of motor behavior was not observed following treatment of 1-year-old MLD mice. Effective treatment of MLD may require that gene therapy be started before the onset of neurological symptoms, or perhaps using a strategy that achieves more widespread transduction within the brain. Combined intrathecal and intraventricular injection of AAV vectors may be such an approach to treating MLD mice already exhibiting neurological symptoms.

## Materials and methods

### Animals

ARSA knockout (MLD) mice were a gift from the laboratory of Dr. V. Gieselmann^43^. These mice have been extensively characterized and are widely used as a mouse model of MLD^44,45^. Mice were housed under a 12 h light–dark cycle and had ad libitum access to food and water. All animal experiments were performed in accordance with the regulations of the Ethics Committee of Nippon Medical School and the ARRIVE guidelines. All experimental protocols were approved by the Ethics Committee of Nippon Medical School (Approval No.: 2020-013).

### Generation of AAV vectors

The recombinant AAV vector plasmid pAAV/GFP, which contains the enhanced GFP gene driven by the CAG promoter, was described previously^46^. The recombinant AAV vector plasmid pAAV/ARSA, which contains cDNA encoding flag-tagged ARSA driven by the CAG promoter as the first gene and GFP cDNA driven by the B19 promoter as the second gene, has also been described previously^16^. AAV serotype 9 packaging plasmids (p5E18VD2/9) were kindly provided by Dr. J. Wilson^47^. Recombinant AAV vectors were generated using a three-plasmid co-transfection system (vector plasmid, packaging plasmid and helper plasmid (pHelper; Stratagene, La Jolla, CA)) and then purified using ammonium sulfate precipitation and iodixanol continuous gradient centrifugation, as described previously^14,48^. The titer of each AAV vector was determined using real-time PCR (7500 Fast, Applied Biosystems, Tokyo, Japan). The approximate titer of the final preparation of each AAV vector was 4.0 × 10^13^ vector genomes (vg)/ml.

### Intrathecal injection

Six-week-old or 1-year-old MLD mice were deeply anesthetized using pentobarbital, placed in a stereotactic frame and flexed in a prone position, after which the back of the neck was cleaned with povidone-iodine. A skin incision was then made at the back of the head for intrathecal injection into the suboccipital cisternal space. The dura mater was exposed between the occipital bone and cervical vertebrae, and a needle was inserted into the cistern via a suboccipital puncture. Ten microliters of vector solution containing 4.0 × 10^11^ vg AAV particles were delivered over a period of 1 min using a Hamilton syringe with a 33-g bevel-tipped needle (Hamilton, Reno, NV)^19,49^. These injection steps were performed manually.

### Immunohistochemical analysis

Immunohistochemical analysis of neuronal cells was performed as described previously^28^. Mice were anesthetized and perfused with phosphate-buffered saline (PBS). The brains and spinal cords were then dissected out, fixed in 4% paraformaldehyde overnight at 4 °C, and immersed in PBS containing 20% sucrose. Thereafter, the specimens were placed in OCT compound (Tissue-Tek, Tokyo, Japan), and tissue sections were cut to a thickness of 20 µm and mounted on glass slides. The sections were then immunostained for ARSA, neurons, cholinergic neurons, astrocytes and GFP by incubation for 16 h at 4 °C with goat anti-ARSA (AF2485; 5 μg/ml, R&D, Minneapolis, MN), mouse anti-NeuN (MAB377; 1:500, Millipore, Billerica, MA), rabbit anti-calbindin D-28K (AB1778; 1:1000, Millipore), mouse anti-parvalbumin (P3088; 1:2000, Sigma-Aldrich, St Louis, MO), goat anti-choline acetyltransferase (ChAT) (AB144P; 1:100, Millipore), mouse anti-GFAP (G3893; 1:500, Sigma-Aldrich), rabbit anti-Iba1 (019-19741; 1:500, FUJIFILM, Osaka, Japan), rabbit anti-human Olig2 (18953; 1:200, IBL, Gunma, Japan), and rabbit anti-GFP (A21311; 1:1000, Invitrogen, Life Technologies Corp., Carlsbad, CA) or rat anti-GFP (04404-84; 1:1000, Nacalai tesque, Kyoto, Japan) antibodies. After washing the sections three times with PBS, they were stained with Alexa Fluor 568-conjugated anti-mouse, anti-rabbit or anti-goat IgG (1:500, Invitrogen) or with Alexa Fluor 488-conjugated anti-rabbit IgG or anti-rat IgG (1:500, Invitrogen). Finally, the sections were examined under a BX60 microscope (Olympus, Tokyo, Japan).

### Determination of ARSA concentrations

Each organ of interest was homogenized in pure water, after which the human ARSA concentration was determined using an indirect sandwich enzyme-linked immune sorbent assay (ELISA), as described previously^16^. Human ARSA does not cross-react with murine ARSA^50^.

### Droplet digital PCR (ddPCR) analysis for detection of vector genome

Total DNA was isolated from AAV/ARSA-transduced mouse tissues using a DNeasy Tissue Kit (QIAGEN, Hilden, Germany), after which the DNA was subjected to droplet digital PCR to detect the copy number of the AAV vector. The mouse haploid genome single-copy gene transferrin receptor (TFRC) (4458366: Thermo Fisher scientific) was selected as an internal control. Each PCR reaction was run in a 20-μL reaction mixture containing 10 μL of ddPCR supermix for probes (Bio-Rad), 900 nM primers, 250 nM probe and 4 μL of template DNA. Droplets were generated on a Bio-Rad QX-200 following the manufacturer’s instructions. Samples were transferred to a 96 well-plate and thermal cycled to the endpoint (T100 Thermal Cycler; Bio-Rad) using a standard protocol: initial denaturation at 95 °C for 10 min followed by 40 cycles of melting at 94 °C for 30 s and annealing/elongation at 55 °C for 1 min before droplet enzyme deactivation by incubation for 10 min at 98 °C. The PCR plate was subsequently scanned using a QX200 droplet reader (BIO-RAD), and the data were analyzed using QuantaSoft software (BIO-RAD). For 2D ddPCR, two sets of primers and probes labeled with FAM and VIC, respectively, were added to each reaction mixture, and ddPCR was performed in the same tube. The following primers and probe were used for ddPCR: 5′-TACATCAAGTGTATCATATGCCAA-3′ (CAG probe), 5′-CAATGGGTGGAGTATTTACG-3′ (forward primer), and 5′- GGTCATGTACTGGGCATAAT-3′ (reverse primer). TaqMan™ copy number reference assay (mouse, Tfrc) was used as an internal control. The vector genome were showed CAG/Tfrc ratios.

### Alcian blue staining and analysis of Sulf and GalC

To detect the accumulation of sulfatides, brain and spinal cord sections were stained with alcian blue (Sigma-Aldrich) as described previously^51^ and quantified in three random areas of each sections using Image J software. Moreover, sulfatide levels were determined using thin-layer chromatography as follows. The excised brains, separated into two segments (forebrain and hindbrain comprising the brainstem and cerebellum), and spinal cords were homogenized in pure water, after which the homogenates were centrifuged at 15,000 rpm for 10 min at 4 °C. The supernatants were then collected for determination of the protein content using a Bio-Rad DC Assay (Bio-Rad, Hercules, CA) and sulfatide content using thin-layer chromatography. Crude lipids were extracted from the tissue homogenates using the Folch method^52^. The levels of sulfatide and galactosylceramide present were determined by densitometric scanning using a DT-20 MCP (Atto, Tokyo, Japan), as described previously^10,28^. Amounts were quantitatively determined with densitometric scanning using CS Analyzer 3 (ATTO, Tokyo, Japan). Data are expressed as the mean ± standard error (SE) of Sulf/GalC ratios.

### Behavior test

Motor coordination and balance were assessed in the mice by measuring their ability to traverse a graded series of narrow beams to reach an enclosed safety platform as described previously^53^. The beams consisted of long (1 m) stainless steel rods, starting at a cross-sectional diameter of 20 mm (O’HARA & Co., Ltd., Tokyo Japan). The beams were placed horizontally, 50 cm above the bench surface, with one end mounted on a narrow support and the other end attached to an enclosed box (20 cm^2^), into which a mouse could escape. The mice were trained to traverse the beam to the enclosed box. The time required to traverse each beam (“latency”) and the number of times the hind feet slipped off a beam (“slips”) were recorded for each trial. Analysis of each measure was based on the mean scores from three trials.

### Statistical analysis

Results are expressed as means ± SE of duplicate or more data obtained from three or more independent experiments. Data were analyzed using One-way ANOVA (GraphPad Prism software, San Diego, CA). Values of p < 0.05 were considered significant.

## Supplementary Information

Supplementary Information.
