# Supplementary material for: Treatment of adult metachromatic leukodystrophy model mice using intrathecal administration of type 9 AAV vector encoding arylsulfatase A
Source: Sci Rep. 2021 Oct 15;11:20513. doi: 10.1038/s41598-021-99979-2 (PMC8521568; doi:10.1038/s41598-021-99979-2)
Supplement: Supplementary file 1 — Supplementary Information. [file 41598_2021_99979_MOESM1_ESM.pdf]

Treatment of adult metachromatic leukodystrophy model mice using intrathecal administration of type 9 AAV vector encoding arylsulfatase A

Noriko Miyake[1], Koichi Miyake[2], Atsushi Sakai[3], Motoko Yamamoto[1], Hidenori Suzuki[3], Takashi Shimada[1]

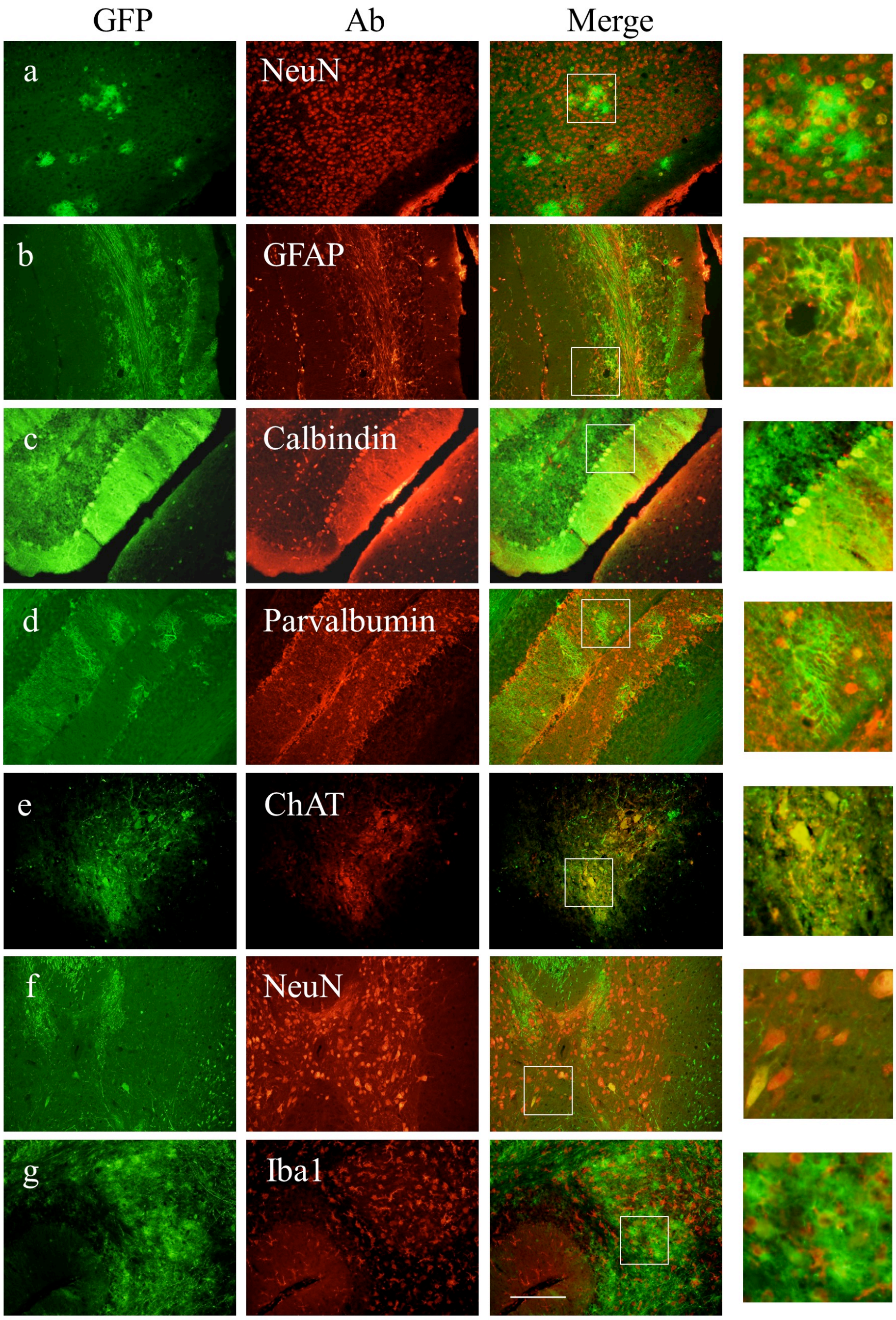

Supplementary Figure S1. Immunohistochemical analysis of transduced cells in the brain and spinal cord of treated MLD mice at 6 week of age

Immunostaining for GFP and the neuronal marker NeuN in the striatum (a), the astrocyte marker GFAP (b), the Purkinje marker calbindin (c), the basket marker parvalbumin in the cerebellum (d), the microglia marker Iba1 (g) in the brain, and ChAT-positive motor neurons (e) and NeuN-positive neurons (f) in the cervical spinal cord. Bar: 200 µM. Panels on the right highlight the observed co-localization.

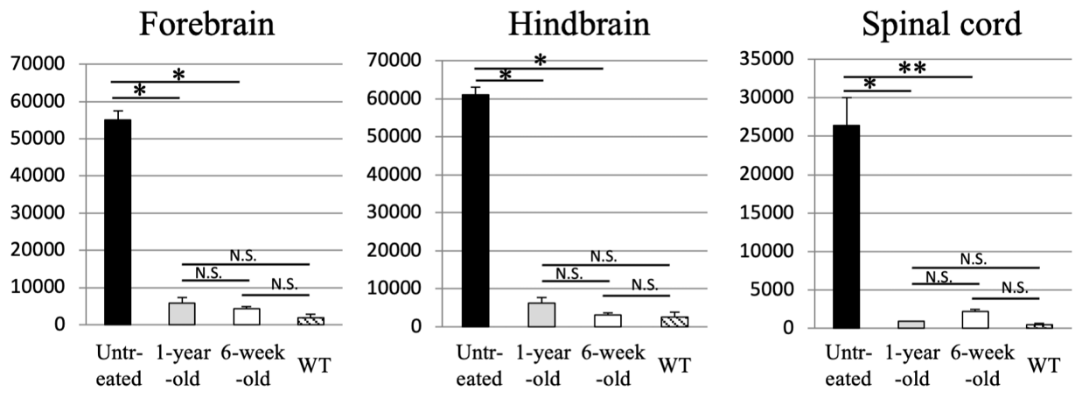

Supplementary Figure S2. Correction of sulfatide storage in the brain and spinal cord. Quantified of sulfatide storage per mm^2 in the forebrain, hindbrain and cervical spinal cord of MLD mice left untreated (closed bars, n =5), MLD mice treated at 1 year (gray bars, n =5) or 6 weeks of age (open bars, n=4), and wild-type (WT) mice (diagonally hatched bars, n=8).
* $P<0.0001$, ** $p<0.003$

Supplementary Table S1. Raw data calculated from thin layer chromatography plates

Forebrain				Hindbrain				Spinal cord			
	Sulf[a]	GalC[b]	Sulf/GalC		Sulf	GalC	Sulf/GalC		Sulf	GalC	Sulf/GalC
Untreated-1	23884	25816	0.925163	Untreated-1	22148	27188	0.814624	Untreated-1	5017	12151	0.412888
Untreated-2	17388	22400	0.77625	Untreated-2	15176	20832	0.728495	Untreated-2	4321	11484	0.376263
Untreated-3	24600	27330	0.90011	Untreated-3	31744	45105	0.70378	Untreated-3	3538	9599	0.36858
Untreated-4	28050	31620	0.887097	Untreated-4	28055	49600	0.565625	Untreated-4	5278	11513	0.458438
Untreated-5	15750	20640	0.763081	Untreated-5	22940	36394	0.630324	Untreated-5	3596	9512	0.378049
Untreated-6	23280	24630	0.945189	Untreated-6	17205	29481	0.583596	Untreated-6	4350	12847	0.3386
1-year-old-1	17976	23632	0.760664	1-year-old-1	17416	27468	0.634047	1-year-old-1	3306	14094	0.234568
1-year-old-2	19488	23548	0.827586	1-year-old-2	15148	29652	0.510859	1-year-old-2	3219	13659	0.235669
1-year-old-3	16296	23380	0.697006	1-year-old-3	11648	22036	0.52859	1-year-old-3	4582	15950	0.287273
1-year-old-4	16352	24332	0.672037	1-year-old-4	13524	26544	0.509494	1-year-old-4	2581	10005	0.257971
1-year-old-5	18060	23070	0.782835	1-year-old-5	15988	27216	0.587449	1-year-old-5	1595	6873	0.232068
6-week-old-1	17280	23640	0.730964	6-week-old-1	23808	41509	0.573562	6-week-old-1	2059	9396	0.219136
6-week-old-2	15720	25350	0.620118	6-week-old-2	20181	38936	0.518312	6-week-old-2	3538	12325	0.287059
6-week-old-3	16140	21420	0.753501	6-week-old-3	12431	29791	0.417274	6-week-old-3	1740	6525	0.266667
6-week-old-4	8820	16950	0.520354	6-week-old-4	9300	30256	0.307377	6-week-old-4	1044	4263	0.244898
WT-1	806	3720	0.216667	WT-1	4710	16290	0.289134	WT-1	1376	4478	0.30730
WT-2	992	3751	0.264463	WT-2	5550	17790	0.311973	WT-2	1073	3775	0.28409
WT-3	1023	3844	0.266129	WT-3	5700	19500	0.292308	WT-3	941	3376	0.27859
WT-4	1116	4433	0.251748	WT-4	3900	16800	0.232143	WT-4	818	2970	0.27556
WT-5	1178	4712	0.25	WT-5	4320	15840	0.272727	WT-5	970	3198	0.30341
WT-6	899	5487	0.163842	WT-6	4768	13472	0.353919	WT-6	835	2858	0.29215
WT-7	806	5053	0.159509	WT-7	4832	17312	0.279113	WT-7	1089	3713	0.29333
WT-8	1085	5332	0.203488	WT-8	6240	24224	0.257596	WT-8	1096	3356	0.32645
WT-9	1457	5332	0.273256					WT-9	805	2878	0.27982
WT-10	1271	4557	0.278912					WT-10	1003	3524	0.28464

[a]Sulf, sulfatide; [b]GalC, galactosylceramide;
